# Short-Term Effects of Air Pollution on Coronary Events in Strasbourg, France—Importance of Seasonal Variations

**DOI:** 10.3390/medsci8030031

**Published:** 2020-08-07

**Authors:** Baptiste Vaudrey, Marie Mielcarek, Erik Sauleau, Nicolas Meyer, Benjamin Marchandot, Marie Moitry, Pierre Robellet, Thierry Reeb, Laurence Jesel, Patrick Ohlmann, Thomas Bourdrel, Olivier Morel

**Affiliations:** 1 Pôle d’Activité Médico-Chirurgicale Cardio-Vasculaire, Nouvel Hôpital Civil, Centre Hospitalier Universitaire, Université de Strasbourg, 67091 Strasbourg, France; benjamin.marchandot@gmail.com (B.M.); laurence.jesel-morel@chru-strasbourg.fr (L.J.); patrick.ohlmann@chru-strasbourg.fr (P.O.); olivier.morel@chru-strasbourg.fr (O.M.); 2 GMRC, Service de Santé Publique, Centre Hospitalier Universitaire de Strasbourg, 67091 Strasbourg, France; marie.mielcarek@chru-strasbourg.fr (M.M.); Erik.SAULEAU@chru-strasbourg.fr (E.S.); nicolas.meyer@chru-strasbourg.fr (N.M.); 3 Department of Public Health, Strasbourg University Hospital, 67091 Strasbourg, France; mmoitry@unistra.fr; 4 Pôle exploitation—Unité Surveillance réglementaire, ATMO Grand EST; association à but non lucratif agréée par le Ministère chargé de l’environnement, 67300 Schiltigheim, France; pierre.robellet@atmo-grandest.eu; 5 Clinique Rhéna, 67000 Strasbourg, France; t.reeb@medecin-alsace.fr (T.R.); thomasbourdrel@yahoo.fr (T.B.); 6 UMR INSERM 1230 Regenerative Nanomedicine, University of Strasbourg, 67000 Strasbourg, France

**Keywords:** air pollution, acute coronary syndrome, NO_2_, PM_2.5_, PM_10_, seasonality

## Abstract

The aim of this study, is to investigate the effects of a short-term exposure to air pollutants, as assessed by Nitrogen dioxide (NO_2_), Particulate Matter PM_2,5_ and PM_10_ concentrations, on coronary event onsets in Strasbourg, France. An observational, analytical, retrospective, epidemiological study was conducted in Strasbourg between 1 January 2012 and 31 December 2014. Higher daily coronary events rates were evidenced when NO_2_ concentrations were measured above 40 µg/m^3^ (1.258 (95% CI 1.142–1.374) vs. 1.110 (95% CI 1.033–1.186); *p* = 0.015). The NO_2_ concentration was higher than 30 µg/m^3^ for 677 days (61.8%). Higher daily coronary events rates were evidenced when NO_2_ concentrations were measured above 30 µg/m^3^ (1.208 (95% CI 1.128–1.289) vs. 1.067 (95% CI 0.961–1.172) *p* = 0.009). A marked seasonality of NO_2_, PM_2.5_, and PM_10_ concentrations characterized by an increase during winter and a decrease during the summer could be established. The seasonality of coronary events was evidenced simultaneously. After adjustments were made to account for the time and the month, no independent impact of NO_2_, PM_2.5_ or PM_10_ on daily coronary events could be demonstrated.

## 1. Introduction

Although numerous epidemiological studies have uncovered noxious associations between the air pollution and the cardiovascular mortality burden, the recognition of air pollution as a silent killer remains largely unknown by the cardiology community. Ref. [1,2,3] Various projections have estimated that outdoor air pollution may be responsible of 3.3 million premature deaths per year worldwide, predominantly in Asia. Ref. [4,5] While the relationship between air pollution and the atherothrombotic burden appears to be likely, the precise mechanisms underlying their association are not fully understood. Previous data has underlined that the air pollution could be associated with key markers of the atherothrombotic burden, including systemic inflammatory response, oxidative stress, endothelial dysfunction, blood coagulation that could pave the way for plaque rupture, and thrombus formation. Air pollution can be divided into two types: gaseous substances and materials particulate. The main gases studied herein are nitrogen oxides (NO_x_) including nitrogen dioxide (NO_2_) and nitrogen monoxide (NO), carbon monoxide (CO), and ozone (O_3_). The main source of NO_x_ emissions is the combustion of fossil fuels. Road transport remains an important source of NO_x_ production worldwide, especially when diesel vehicles are used. Particles are classified according to their aerodynamic diameter. The usual distinction was made between the “coarse” particles called PM_10_ (diameter < 10 μm), PM_2.5_ or fine particles (diameter < 2.5 μm), and ultrafine particles also called nanoparticles (PM_0.1_, diameter < 0.1 μm). The sources of anthropogenic emissions of PM_10_ and PM_2.5_ lie with various sectors including the agricultural, the residential, the tertiary, the industrial, and the transport sectors. While a minority of studies suggests an increase in the risk of acute coronary syndromes following exposure to elevated O_3_ concentrations, the impact of short-term exposure to NO_2_, PM_2.5_, and PM_10_ on myocardial risks appears to be more consistent [6,7,8,9,10,11]. For example, Bhaskaran et al. in a meta-analysis have noticed that PM_2,5_ was associated with an incremental risk of myocardial infarction (from 5% to 17% per 10 µg/m^3^ increase), PM_10_ was associated with an incremental risk of myocardial infarction (0.7–11% per 10 µg/m^3^) and NO_2_ also (1–9% per 10 ppb). In order to protect vulnerable populations, various warning thresholds have been defined by regulatory agencies. For example, European recommendations indicate that the annual NO_2_ rate should not exceed 40 µg/m^3^. The recommendation and information threshold for the population is triggered when NO_2_ rate is greater than or equal to 400 µg/m^3^ on an hourly average, for 3 consecutive hours.

For PM_10_, the annual average not to be exceeded is 40 µg/m^3^. for PM_2.5_, the annual average not to be exceeded is 25 µg/m^3^.

However, these recommendation and information threshold appears to be very high and is not encountered frequently in daily practice in European countries.

In the present study, we aim to investigate the effect of short-term exposure of air pollutants as assessed by Nitrogen dioxide (NO_2_), Particulate Matter (PM) in PM_2.5_ and PM_10_ concentrations on coronary event onsets in Strasbourg, France. In addition, seasonality of air pollution and of coronary events is also studied.

## 2. Materials and Methods

### 2.1. Population Studied

The study was restricted to the inhabitants of Strasbourg. In 2014, Strasbourg had 276,170 inhabitants in an area spanning 78.3 km^2^. Ref. [12] Strasbourg city is located in the North East of France (“Region Grand Est”), close to the German border (latitude 48.5734053, longitude 7.7521113).

### 2.2. Cases

The cases were defined as coronary events, either fatal or non-fatal, in men and women, occurring in the age group of 35–74 years, between 1 January 2012 and 31 December 2014. These cases were identified by the MONICA registry [13]. To safeguard the anonymity of the patients involved, no meaningful clinical information (sex categories, age, cardiovascular risk factors, etc.) could be recorded. There were no exclusion criteria.

### 2.3. Assessment of Exposure to Air Pollution

Data on daily air pollution was collected from ASPA (now called «Atmo Grand Est»), which is a non-profit organization labelled by the Ministry of the Environment, which is in charge of air quality monitoring in the «Region Grand Est». Daily air pollution was assessed using 24 h average NO_2_, PM_10_, and PM_2.5_ concentrations. Three fixed stations in Strasbourg city were used to measure the air pollution. Strasbourg North: measurement of NO_2,_ PM_10_; Strasbourg East: measurement of NO_2_ and PM_2.5_; Strasbourg CLEMENCEAU (Traffic zone): measurement of NO_2_ and PM_10_. For simplicity’s sake, the daily measurements of NO_2_ in the stations North, East and CLEMENCEAU were averaged. Similarly, the daily measurements of PM_10_ in the stations North and East were averaged as well.

### 2.4. Seasonality

Strasbourg city has a semi-continental climate with a warm summer (July 19.2 °C) and a cold winter (January 0.9 °C). Four time periods were considered: winter for the months of January, February, March; spring for the months of April, May, June; summer for the months of July, August, September; and fall (autumn) for the months of October, November, and December.

### 2.5. Lags

As plaque rupture can cause immediate or sometimes delayed clinical manifestations of acute coronary syndromes due to dynamic thrombotic response, several lags between air pollution and myocardial infarction (MI) events were investigated: the average of the day preceding the coronary event (lag Day-1), the average of the day which preceded the coronary event by 2 days (lag Day-2), the average of the day which preceded the coronary event by 3 days (lag Day-3).

### 2.6. Statistical Analysis

This was an epidemiological, observational, analytical, and retrospective study. The descriptive statistical analysis of the quantitative variables was done by indicating the position parameters (average, median, minimum, maximum, first, and third quartiles), as well as the dispersion parameters (variance, standard deviation, range, interquartile range) for each variable. The Gaussian character of the data was tested by the Shapiro–Wilk test. The description of the qualitative variables was done by providing the numbers and proportions of each category in the sample. The comparison of quantitative variables between groups was performed either by a Student’s test (when the variable of interest was Gaussian) or by a nonparametric test in the opposite case (Mann-Whitney-Wilcoxon test). Generalized additive models (GAM) were used for the inferential analysis. They make it possible to process count data and to produce a smoothing of the effect of the explanatory variables.

## 3. Results

### 3.1. Number and Categories of Coronary Events

In total, 1265 coronary events were recorded between 1 January 2012 and 31 December 2014 (1096 days). The daily coronary event rate was 1.154/day on average. Diagnostic categories of coronary events and the daily repartition are shown in Table 1 and Table 2.

### 3.2. Pollutants

The air pollution data is illustrated in Table 3. Mean and range of the air pollutant studied in Strasbourg was comparable to that shown in a previous report made in the same Strasbourg Metropolitan Area [14].

Only very little data was missing (NO_2_: 0%, PM_2.5_: 5.47% (60 days), PM_10_: 0.27% (3 days)).

### 3.3. Impact of the NO_2_ Concentration on Coronary Events

The NO_2_ concentration was higher than 40 µg/m^3^ on 329 days (30%). Higher daily coronary events rates were evidenced when NO_2_ concentrations were measured above 40 µg/m^3^ (1.258 (95% CI 1.142–1.374) vs. 1.110 (95% CI 1.033–1.186); *p* = 0.015). The NO_2_ concentration was higher than 30µg/m^3^ on 677 days (61.8%). Higher daily coronary events rates were evidenced when NO_2_ concentrations were measured above 30µg/m^3^ (1.208 (95% CI 1.128–1.289 vs. 1.067 (95% CI 0.961–1.172) *p* = 0.009).

### 3.4. Impact of the PM_2.5_ Concentration on Coronary Events

The PM_2.5_ concentration was higher than 25 µg/m^3^ on 191 days (17.4%). No impact of a PM_2.5_ concentration above 25 µg/m^3^ on the daily coronary event rate could be established (>25 µg/m^3^: 1.168 (95% 1.031–1.304) vs. <25 µg/m^3^: 1.151 (95% CI 1.079–1.223); *p* = 0.407).

### 3.5. Impact of the PM_10_ Concentration on Coronary Events

The PM_10_ concentration was higher than 40 µg/m^3^ on 123 days (11.2%). No impact of a PM_10_ concentration above 40 µg/m^3^ threshold on the daily coronary event rate could be established (>40 µg/m^3^: 1.114 (95% 0.962–1.266) vs. <40 µg/m^3^: 1.160 (95% CI 1.090–1.230); *p* = 0.950).

### 3.6. The Seasonality of Coronary Events

**Figure 1:**
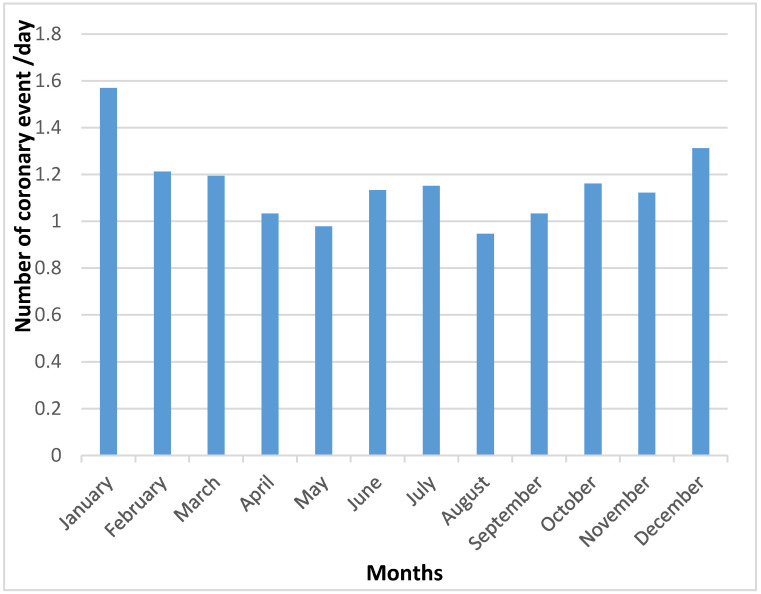
Daily coronary event rate for each month.

**Figure 2:**
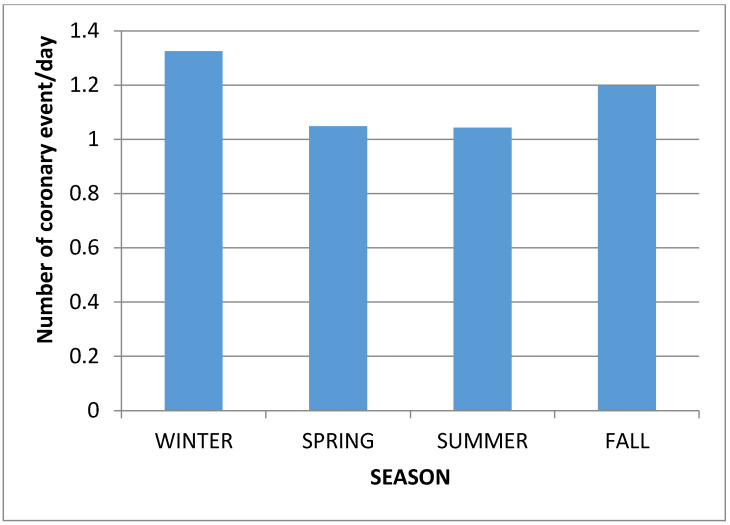
Daily coronary event for each season.

The daily coronary event rate was collected and analyzed for each month (Figure 1) and for each season (Figure 2), between 1 January 2012 and 31 December 2014.

The incidence of coronary events was at its highest level in the winter (January) and at its lowest level in the summer (August) (*p* = 0.009) (Figure 1 and Figure 2).

### 3.7. Inferential Analysis: Generalized Additive Model (GAM)—Pollutant and Time Effect

**Figure 3:**
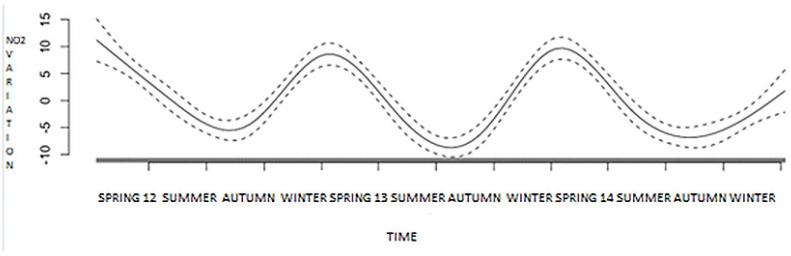
Time effect on NO_2_ concentrations.

In order to address the effect of time on the concentrations of pollutant, an inferential analysis using a generalized additive model was performed. A clear seasonality of air pollutant concentration could be evidenced, with a decrease in the summer and increase in the winter. Air pollutants were at their highest levels in January and at their lowest levels in August (*p* < 0.001). Similar variations were evidenced for NO_2_ (Figure 3), PM_2.5_ and PM_10_.

When a time adjusted analysis was performed, no independent impact of NO_2,_ PM_2.5_, and PM_10_ concentrations on daily coronary events could be demonstrated.

No relationship between different intervals (Day-1, Day-2, Day-3) of air pollutant concentration (NO_2_, PM_2.5_, PM_10_) and the onset of MI could be evidenced.

## 4. Discussion

In the present study, the effect of a short-term exposure to air pollutants as assessed by NO_2_, PM_2.5_, and PM_10_ concentrations on coronary event onsets was examined in an urban population located in Strasbourg, France.

In Strasbourg, a continuous decline in the annual mean concentrations of NO_2_ can be evidenced since 2005. In 2013, road transport was still the leading cause of NO_x_ emissions (58%), although a significant decrease (by 44%) of its emissions has been evidenced since the early 2000s. Possible explanations could rely on a continuous renewal of the vehicle fleet despite a significant increase in heavy goods vehicle traffic along the main highways [15]. Additionally, specific City Hall policies were developed over time to limit air pollution. For instance, a public transport network including high-frequency bus and tramway was developed, and Strasbourg had the largest cycle path network in France for many years (more than 600 kms/373 miles). Despite continuous efforts, the inhabitants of Strasbourg were exposed to significant levels of the NO_2_ concentration (>40 µg/m^3^) every third day. In this metropolitan area, higher daily coronary events were recorded when NO_2_ concentrations peaked above 40 µg/m^3^. Importantly the association between daily coronary events and NO_2_ concentrations was even evidenced at a lower threshold (>30 µg/m^3^), which was usually considered to be safe. In Europe, different thresholds for NO_2_ concentrations are implemented. The mean value of NO_2_, as an annual mean, should not exceed 40 µg/m^3^, whilst the 200 µg/m^3^ threshold should not be exceeded for more than 18 h in a year. Warning information should be triggered when NO_2_ peaks above 400 µg/m^3^ for more than 3 consecutive hours. In the present study, the threshold of 200 µg/m^3^/h has been exceeded on 23 days (14 days in 2012, 6 days in 2013, 3 days in 2014). Important controversies remain on the independent impact of air pollution on coronary events. For instance, in the large England and Wales Myocardial Ischemia National Audit project gathering 202 550 STEMI (ST Elevation Myocardial Infarction) and 322 198 NSTEMI (Non-ST Elevation Myocardial Infarction), no association between NO_2_ and STEMI could be established. Conversely, in NSTEMI, a positive association between daily NO_2_ concentration and NSTEMI could be demonstrated that persisted following adjustment for O_3_ and PM_2.5_. In this trial, a 0.27% increase in risk per 10 µg/m^3^ increase in NO_2_ concentration could be established [11]. Accordingly, in the work by Milojevic and coworkers, positive association between NO_2_ (lags 0–4) with NSTEMI could also be demonstrated, with a more pronounced estimated risk (0.68% per 10 µg/m^3^ increase) [16]. Another recent report has underlined a noxious synergy between road traffic noise and NO_2_ with a prominent role for traffic noise [17]. Other meta-analyses have emphasized the view that NO_2_ concentrations were closely associated with hospital admissions or stroke mortality [18]. Up to now, only few reports have explored the pathological pathways linking NO_2_ exposure to plaque rupture and thrombus formation. In the work by Mills, exposure of healthy volunteers to air pollutant including NO_2_ induced a swift impairment of endothelial function and endogenous fibrinolysis [19]. Other pathways include systemic inflammatory response, oxidative stress, and enhanced platelet activation, key mediators in plaque formation, rupture, and subsequent thrombus formation [20,21].

Other data has highlighted the seasonality of air pollution and coronary events [22,23,24,25]. Interestingly, the winter predominance of coronary events was even described in the south hemisphere when winter season corresponds to warmer temperatures. Geographically, Strasbourg is located in the Rhine plane bordered by the Vosges and the Black Forest mountain massifs and is frequently affected by the winter inversion phenomenon. In our study, both coronary events and air pollution peaked during the winter. When time and seasonal adjustments were performed, the independent impact of NO_2_ concentrations on myocardial infarction could not be established. Possible explanations include (i) the limited power of the study (1265 coronary events) when considering the limited specific impact of NO_2_ exposure (0.27 to 0.68% increase per 10 µg/m3 increase), (ii) the collinearity of the data, and (iii) unrecorded confounding factors. Among them, the impact of cold weather conditions, concurrent respiratory infections, the decrease in the number of hours of sunshine, and stress conditions including catecholamine activation possibly involved in the triggering of coronary events deserve further investigation [26].

Limitations of the study: This analysis shares the inherent limitations of any retrospective registry. Demographics and clinical characteristics could not be studied. The coronary event was a composite criterion that comprised 6 categories, including myocardial infarction. STEMI and NSTEMI were not individualized. Individual exposure to air pollution was not assessed. Confounding factors, which possibly explained seasonal variation such as inflammatory markers or concomitant respiratory infections were not investigated. Finally, possible variations in the population during the holidays (especially in summer) were not taken into account.

## 5. Conclusions

In Strasbourg, France, higher daily coronary events were recorded when NO_2_ concentrations peaked above >30 µg/m^3^. After time adjustment, no independent impact of the air pollution on coronary events could be determined. The specific role of cold weather conditions, concurrent respiratory infections, or the decrease in the number of hours of sunshine on coronary events onset deserves further investigation.

## Figures and Tables

**Table 1:** Diagnostic categories of coronary events.

Diagnostic Categories	Number of Coronary Events (% of Total Events)
1 Myocardial infarction characterized	243 (19.2%)
2 Probable coronary death	74 (5.8%)
3 Sudden death in less than 24 h	172 (13.6%)
4 Acute Coronary Syndrome	443 (35%)
5 Unstable angina	159 (12.6%)
6 Other acute form of ischemic heart disease	174 (13.9%)

**Table 2:** Number of daily coronary events.

Daily Coronary Events	Days (%)
0	353 (32.2%)
1	372 (33.9%)
2	261 (23.8%)
3	83 (7.6%)
4	18 (1.6%)
5	6 (0.5%)
6	2 (0.2%)
7	0
8	1 (0.1%)

**Table 3:** Daily concentrations of air pollutants (µg/m^3^).

Pollutant	Mean	SD	Min	Q1	Med	Q3	Max	IQR
NO_2_	34.68	12.53	9.67	25.33	33.33	41.67	92.67	16.33
PM_2.5_	17.49	10.60	2	10	15	22	66	12
PM_10_	23.82	13.30	5,5	14	21.5	30.5	87.5	16.5

SD = standard deviation, Min = Minimum, Q1 = Quartile 1, Med = Median, Q3 = Quartile 3, Max = Maximum, IQR = Interquartile Range.
